# Malignant Pleural Effusion As the Initial Presentation of Renal Cell Carcinoma: A Case Report and Literature Review

**DOI:** 10.7759/cureus.37128

**Published:** 2023-04-04

**Authors:** Mustafa Wasifuddin, Nosakhare Ilerhunmwuwa, Narek Hakobyan, Ephrem Sedeta, Ifeanyi Uche, Henry O Aiwuyo, Jamal C Perry, Omid Heravi, Avezbakiyev Boris

**Affiliations:** 1 Internal Medicine, Brookdale University Hospital Medical Center, Brooklyn, USA; 2 Medicine, Brookdale University Hospital Medical Center, Brooklyn, USA; 3 Oncology, Brookdale University Hospital Medical Center, Brooklyn, USA; 4 Hematology/Oncology, Brookdale University Hospital Medical Center, Brooklyn, USA

**Keywords:** renal cell carcinoma metastasis, pleural biopsy, pleural mass, rare cause of pleural effusion, clear cell renal carcinoma, malignant pleural effusion, renal cell carcinoma (rcc)

## Abstract

Renal cell carcinoma is the most common renal neoplasm. Its presentation is often very occult, and it may be discovered incidentally. It may present with the classic symptoms of back pain, flank pain, hematuria, or hypertension. Renal cell carcinoma may also present with malignant pleural effusion at diagnosis; however, it is very rare. In this case report and literature review, we describe a 77-year-old male who was diagnosed with renal cell carcinoma after presenting with a malignant pleural effusion - an extremely rare phenomenon. An analysis of the literature yielded 13 case reports, including ours, where the diagnostic presentation of renal cell carcinoma was a malignant pleural effusion.

Our patient presented with left-sided chest pain. Imaging was suggestive of pleural effusion. CT and MRI imaging demonstrated masses in the upper and lower poles of the right kidney suggestive of renal cell carcinoma. CT imaging also showed lung nodules that were suggestive of pulmonary metastases. Biopsy and immunostaining of pleural tissue were positive for clear cell renal cell carcinoma. Therapeutic thoracentesis was performed. Despite this, the patient developed recurrent large-volume pleural effusions requiring drainage and placement of a pleural catheter. Our patient’s extremely rare presentation of malignant pleural effusion as the diagnostic presentation of renal cell carcinoma along with recurrent, large-volume effusions requiring drainage has only been reported in the form of case reports in the literature.

## Introduction

Pleural effusions are defined as a build-up of fluid between the pleura and lung parenchyma. They occur secondary to a myriad of pathologies. Malignant pleural effusions are a subset of pleural effusions that may occur secondary to solid tumors like those of the breast and lung [1]. Renal cell carcinoma (RCC) is a solid tumor that may lead to malignant pleural effusion. Only 1%-2% of malignant pleural effusions are secondary to RCC [2]. Due to the highly vascular nature of the tumor, distant site metastasis is very common - with the lung being the most common site. Pleural metastasis is very rare [2].

Very little is reported in the literature on RCC presenting with malignant pleural effusion. The diagnosis of malignant pleural effusion is based on the cytology of fluid obtained from thoracentesis. It may also be obtained from a biopsy of the surrounding tissue, such as the pleura. In RCC, the presence of a malignant pleural effusion signifies stage IV disease and carries a poor prognosis. The five-year survival rate for such patients is 54% for resectable metastases and 29% for non-resectable [3]. Therefore, the goals of therapy for patients who present with stage IV disease such as malignant pleural effusion should prioritize quick symptomatic relief.

## Case presentation

**Figure 1:**
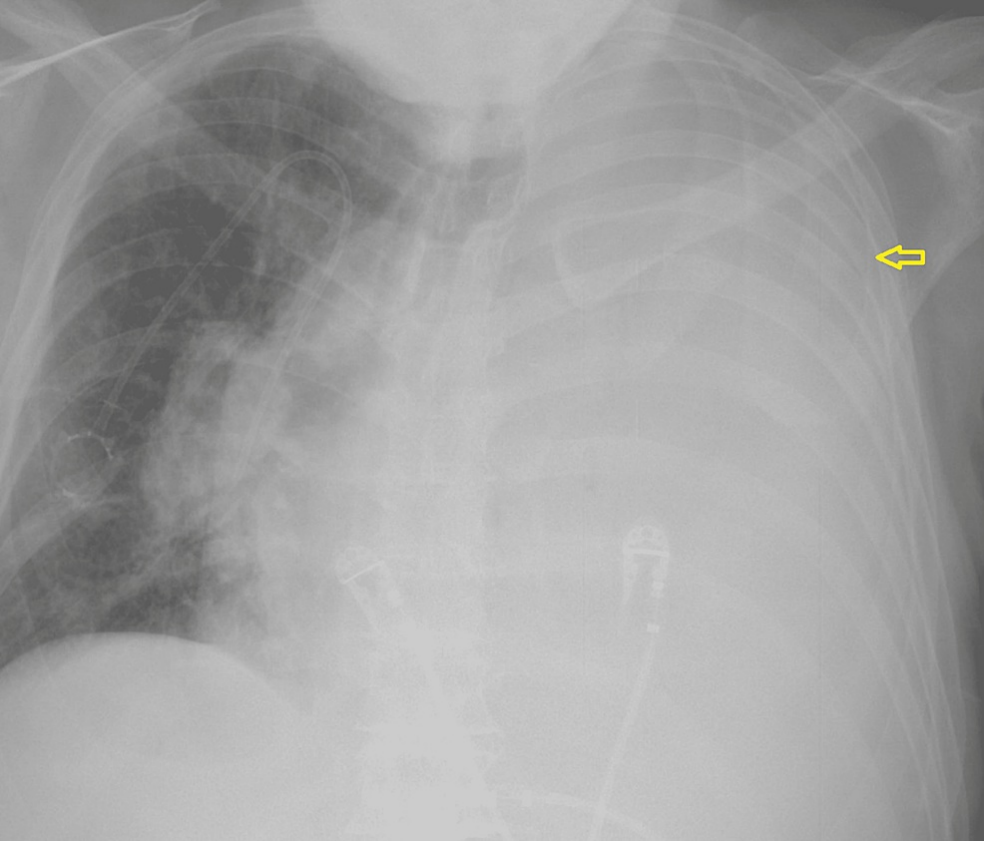
Chest radiograph demonstrating opacification of the entire left hemithroax

**Figure 2:**
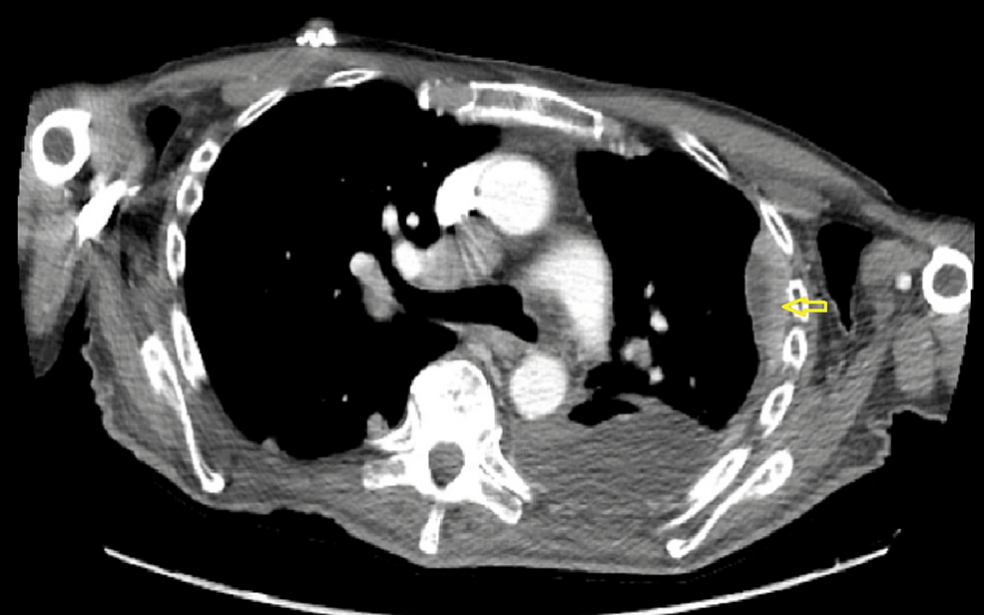
Chest CT demonstrating nodular pleural thickening in the left lung

**Figure 3:**
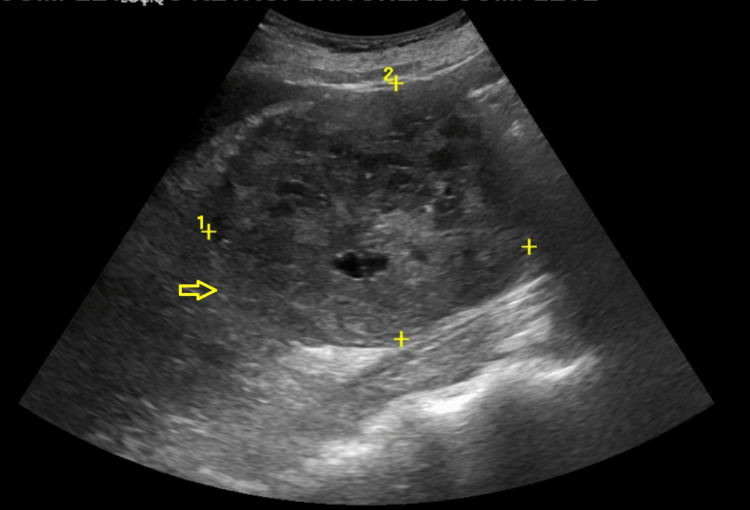
A 10.2 x 8.1 cm heterogeneous partially cystic and partially solid mass (arrow) arising from the lower pole of the left kidney seen on renal ultrasound - suggestive of RCC

**Figure 4:**
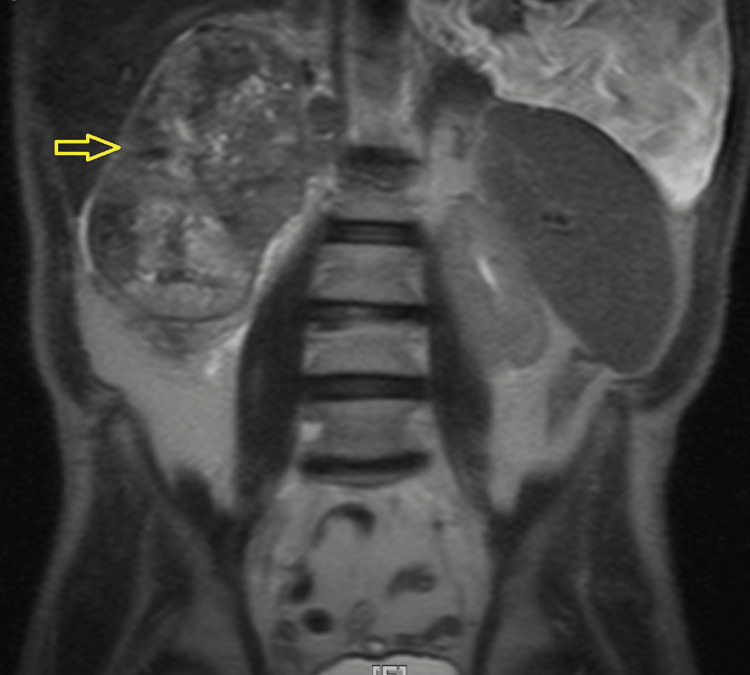
Abdominal MRI demonstrating very large right renal mass encompassing most of the right kidney, concerning for RCC

A 75-year-old male with a history of diabetes mellitus and hypertension was brought in by the EMS for five days of chest pain. The pain was not exacerbated or relieved with any measures. It was not associated with shortness of breath, palpitations, back or flank pain, or hematuria. Lab testing was pertinent for an elevated creatinine of 2.2 mg/dL. A chest x-ray demonstrated extensive opacification of the left hemithorax (Figure 1). Further inquiry of this opacification was done with CT that showed a large left pleural effusion with near complete consolidation of the entire left lung. Sections of the CT scan showed nodular thickening of the pleura (Figure 2). Mediastinal and hilar lymphadenopathy was also noted. A renal ultrasound showed large masses arising from the upper and lower poles of the right kidney suspicious of malignancy (Figure 3). Abdominal MRI showed a large right renal mass encompassing most of the kidney concerning RCC (Figure 4). An ultrasound-guided left pleural mass biopsy was conducted along with left thoracentesis. Biopsy results were positive for metastatic RCC. Pleural fluid analysis was positive for a few small cells with clear cytoplasm but was largely inconclusive. Immunostaining of the pleural tissue was positive for CAIX (diffuse and membranous staining), PAX-8, and Vimentin and negative for CK7 - suggestive of metastatic clear cell RCC.

The patient was diagnosed with Stage IV RCC with lung and pleural metastasis. Of note, ultrasound-guided thoracentesis that was performed earlier 1L of hemorrhagic fluid containing >100,000 RBCs. He was discharged from the hospital and was scheduled to receive outpatient chemotherapy with pembrolizumab and axitinib. Pembrolizumab is a monoclonal antibody directed against PD-1 to restore an anti-tumor response in T-cells. Axitinib is a selective inhibitor of vascular endothelial growth factor receptor (VEGFR) tyrosine kinase.

**Figure 5:**
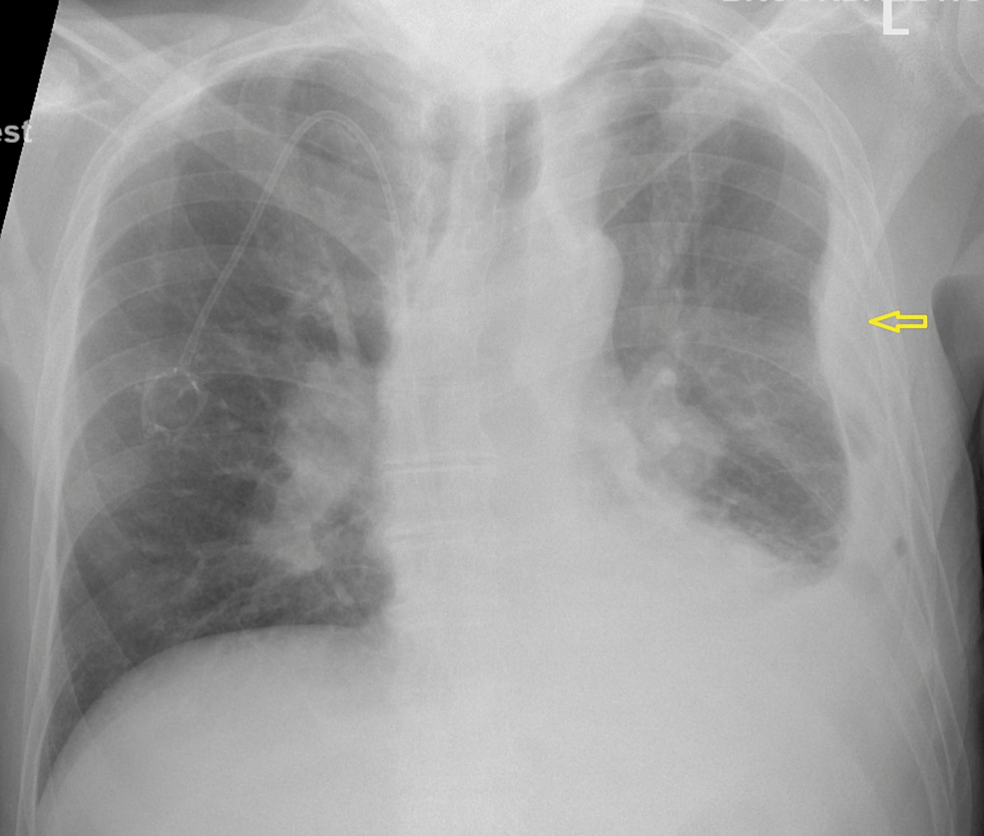
Chest radiograph demonstrating “trapped lung” - a complication of loculated pleural effusion

However, he returned to the ED a few days later after a fall and developing back pain. He also complained of concomitant chest pain. He was noted to be hypoxic with an oxygen saturation of 87% on pulse oximetry. Imaging was suggestive of recurrence of pleural effusion. Thoracentesis yielded 2.5L of serosanguineous fluid. The fluid was drained by thoracentesis and a tunneled catheter was placed in the pleura. It drained a further 2.5L of serosanguineous fluid over the next 24 hours. The patient had continued drainage of fluid after placement of catheter over the next few days. He showed symptomatic improvement with reduction in chest pain and improvement in oxygen saturation on pulse oximetry. A repeat CT scan of the chest and chest x-ray showed a loculated pleural effusion with a trapped lung (Figure 5). Considering the extensive metastases and imaging findings, pleurodesis was not performed for the patient. The patient is scheduled for follow up in the hematology/oncology clinic upon discharge for outpatient chemotherapy.

## Discussion

Renal cell carcinoma’s tendency to present clinically in myriad ways has earned it the name “the internist’s tumor.” It may present with the classic triad of flank pain, hematuria and abdominal mass. More commonly, it presents without any symptoms and is detected incidentally in up to 50% of patients [2]. When complicated with pleural effusion patients may present with chest pain usually accompanied by shortness of breath and hypoxia. Our patient presented with only chest pain due to his underlying effusion. Only 1%-2% of malignant pleural effusions are secondary to RCC, making it a very rare phenomenon [2]. Pleural effusion as the initial presentation of RCC is extremely rare. In most cases, pleural effusion is observed in the diagnosed case of RCC [4]. A literature review by Yasuda et al evaluated seven cases of RCC with localized pleural metastases. The patients in all cases presented with malignant pleural effusions after having been previously diagnosed with RCC [5]. There is a paucity of data in the literature on the incidence of malignant pleural effusion as the initial presentation of RCC, with data primarily existing in case reports. In our case, pleural effusion was the diagnostic presentation of the RCC. Our review of the literature revealed 13 case reports with malignant pleural effusion as the diagnostic presentation of RCC - to the best of our knowledge, making it an extremely rare phenomenon. The cases are listed in Table 1 [2,6-16]. The primary presenting symptom was dyspnea. Five cases had metastases to both lung and pleura, six had isolated pleural metastases, one case had no metastases to the pleura or lung and in one case lung or pleura, involvement was not specified. The mean age for the patients was 53.6 years and the median age was 53 years. All patients were males. The diagnosis was confirmed with pleural fluid cytology, soft tissue biopsy of pleura, immunohistochemistry and renal biopsy. Nine cases were of the clear cell histology, one papillary, one medullary and two were not specified.

**Table 1:** Table demonstrating characteristics of patients with malignant pleural effusion as the diagnostic presentation of the renal cell carcinoma

Patient	Age, Gender	Presenting Symptoms	Lung and pleura involvement	Method of Diagnosis of Renal Cell Carcinoma	Histology	Reference
1	46, M	Dyspnea	Lung, pleura	Pleural biopsy, immunohistochemistry	Clear cell	6
2	66, M	Dyspnea	Pleura	Pleural biopsy	Clear cell	7
3	56, M	Dyspnea, flank pain, back pain	Pleura	Pleural fluid cytology, pleural biopsy, renal biopsy	Clear cell	2
4	34, M	Fever, cough, chills, chest pain and dyspnea	Pleura	Pleural fluid cytology, immunohistochemistry, left nephrectomy and biopsy	Not specified	8
5	53, M	Dyspnea, cough	Lung, pleura	Lung parenchymal biopsy, pleural biopsy, right nephrectomy and biopsy, immunohistochemistry	Clear cell	9
6	81, M	Dyspnea	None	Pleural fluid cytology, left renal biopsy	Clear cell	10
7	50, M	Headache, cough	Pleura	Pleural tissue biopsy	Clear cell	11
8	39, M	Dyspnea	Lung, pleura	Pleural biopsy, immunohistochemistry, partial nephrectomy with biopsy	Clear cell	12
9	67, M	Chest pain, dyspnea	Lung, pleura	Pleura biopsy	Not specified	13
10	53, M	Cough, weight loss	Pleura	Pleura biopsy	Clear cell	14
11	26, M	Fever, cough, dyspnea	Not specified	Pleural fluid cytology, immunohistochemistry, renal biopsy	Medullary	15
12	51, M	Cough, chest pain	Pleura	Pleura soft tissue biopsy, renal biopsy, immunohistochemistry	Papillary	16
13	75, M	Dyspnea	Pleura	Pleura soft tissue biopsy, immunohistochemistry	Clear cell	Present case

Our patient also had recurrent, large volume pleural effusions requiring drainage of more than 5L. The highly vascular nature of RCC can explain the large size of the effusion. The size of effusions and their recurrences have not been well documented in the literature [2]. The presence of large volume effusions in patients with RCC is indicative of an advanced disease stage [2]. The presence of cancer cells in the pleural fluid often confers a poor prognosis. Scoring systems like the LENT prognostic score for malignant pleural effusion can help with prognostication of patients with RCC. LENT stands for - pleural fluid lactate dehydrogenase, eastern cooperative oncology group performance score, neutrophil-to-lymphocyte ratio and tumor type. However, in situations where pleural fluid cytology may be inconclusive, pleural soft tissue biopsy is done to obtain a definitive diagnosis. Our patient was diagnosed with stage 4 clear cell carcinoma after pleural biopsy.

RCC has the potential to seed to distant organs - with lung being the most common site. One study showed that lung metastasis can occur in up to 70% of renal cell carcinoma patients [17]. Pleural involvement is very uncommon. In a study conducted in Japan, pleural involvement was found in up to 12% of autopsied patients [18,19]. Pleural involvement in renal cell carcinoma can be explained by the flow of blood from renal veins into the bronchial and intercostal veins via Batson's plexus and through lymphatic spread [11,19]. Metastases from the kidneys spread to the lung parenchyma, mediastinum and pleura in this manner. Our patient had involvement of both the lung parenchyma and pleura with imaging findings suggestive of lung metastases and biopsy findings suggestive of pleural spread.

**Figure 6:**
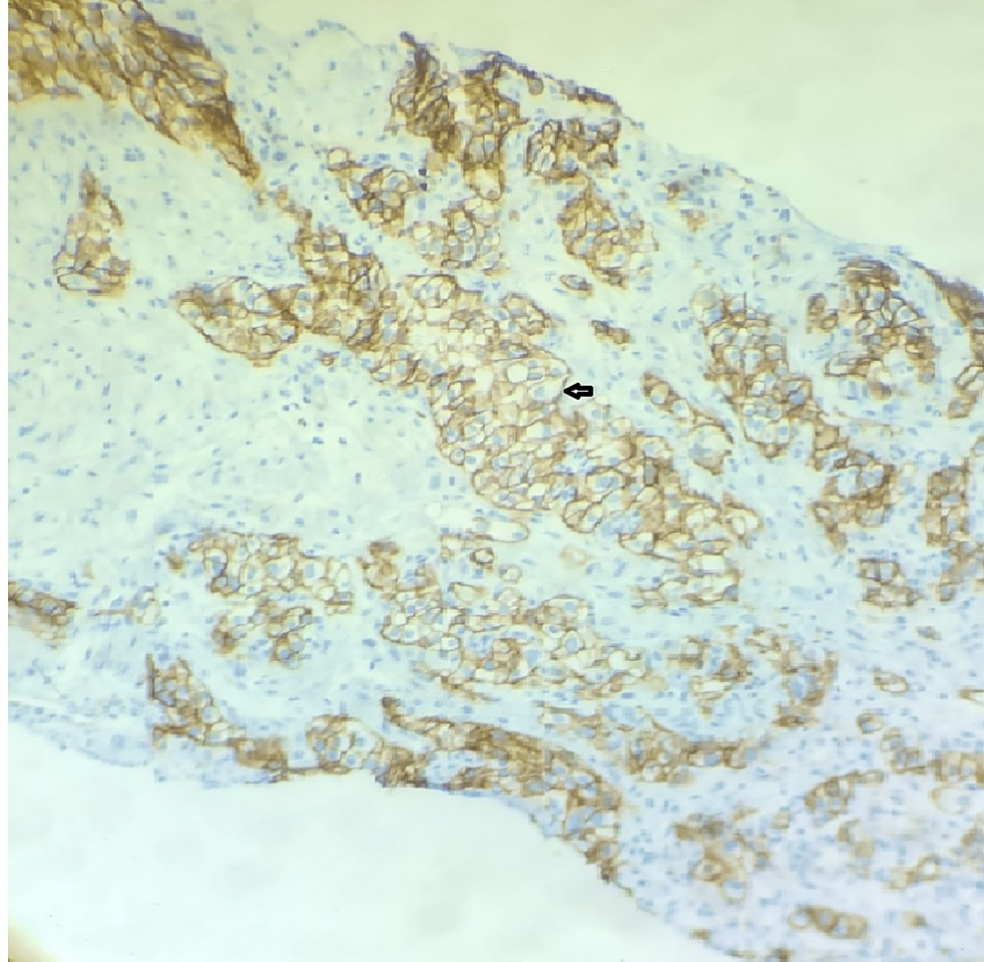
PAX8 staining of pleural tissue showing clear cell adenocarcinoma. Arrow pointing to a clear cell.

**Figure 7:**
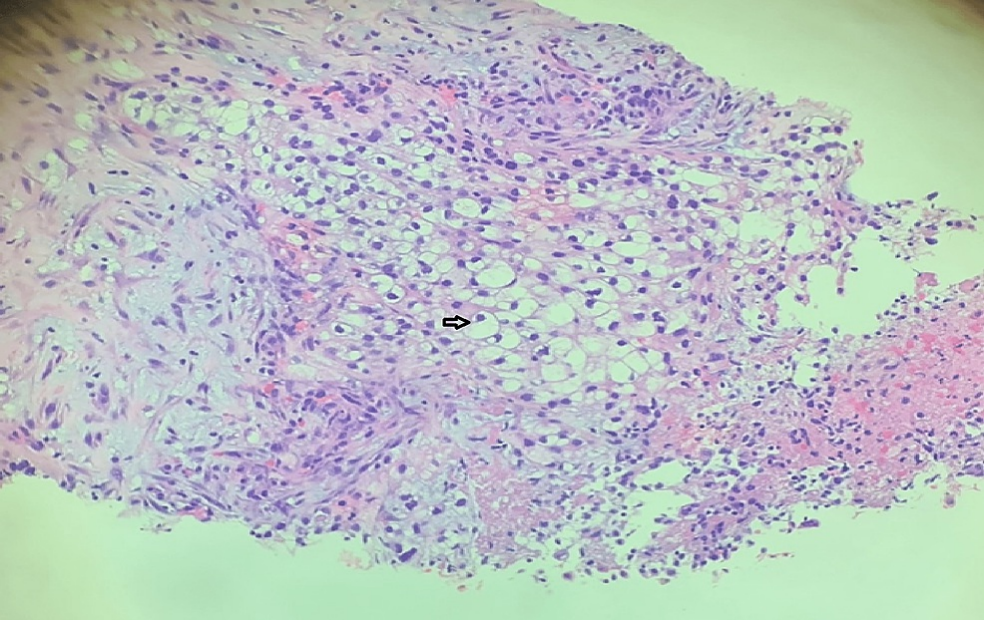
H and E stain of pleural biopsy specimen showing many clear cells. Arrow pointing to a clear cell.

Abdominal computed-tomography (CT) scans and magnetic resonance imaging (MRI) are the commonly used imaging modalities used to detect RCC. Definitive diagnosis is achieved with biopsy of renal tissue or nephrectomy. In our case the diagnosis was suspected after the patient underwent a series of imaging, including chest radiograph, abdominal CT scan and after biopsying pleural tissue. Immunohistochemistry is a powerful tool to help delineate the histopathological subtype of the cancer [20]. Immunostains that are supportive of renal cell carcinoma in a biopsy tissue include CAIX, PAX-8 and Vimentin. Immunostaining of our patient's pleural tissue was positive for CAIX (diffuse and membranous staining), PAX-8 (Figure 6), Vimentin and negative for CK7, confirming the diagnosis of clear cell adenocarcinoma of the kidney (Figure 7).

## Conclusions

Malignant pleural effusions secondary to RCC are a very rare phenomenon. Malignant pleural effusion as the diagnostic presentation of RCC is extremely rare. There have been only 13 cases reported in the literature - to the best of our knowledge, including ours. The presence of malignant pleural effusion in a patient is indicative of an advanced stage of disease (stage 4).

The presentation of RCC as pleural effusion is rare. The presence of recurrent, large-volume effusions may be indicative of an advanced stage of disease, like in the case of our patient. The data for the size of effusions have not been well documented in the literature. A patient presenting with large volume, recurrent pleural effusions should prompt a clinician to include on their differential list malignant pleural effusion secondary to RCC. Since these patients are usually at an advanced stage of the disease the goals of therapy should prioritize quick symptomatic relief.
